# Soft tissue tumor of metastatic non-small cell lung carcinoma: A rare case report

**DOI:** 10.1016/j.ijscr.2024.109621

**Published:** 2024-04-10

**Authors:** Andriandi, C. Tirta

**Affiliations:** a Department of Orthopaedic and Traumatology, Faculty of Medicine Unversitas Sumatera Utara - Adam Malik General Hospital, Medan, Indonesia; b Department of Orthopaedic and Traumatology, Faculty of Medicine Unversitas Sumatera Utara - Adam Malik General Hospital, Medan, Indonesia

**Keywords:** Non-small cell carcinoma, Soft tissue neoplasm, Lung neoplasm, Case report

## Abstract

**Introduction and importance:**

It is estimated that 1 out of 5 patients with cancer will experience bone metastasis. With non-small cell lung cancer by itself having 220.000 reported cases per year, but the prevalence of soft tissue metastasis from lung cancer is only 2.3 % making it commonly overlooked as a possible metastasis site.

**Case presentation:**

Male presents with a lump and pain on the right upper arm. A 8 cm × 8 cm mass was palpated under the biceps. CT-scan showed a lung lesion on the anterior segment. Shoulder MRI showed a dense, lobulated, and indefinitely demarcated soft tissue mass approximately 5.6 cm × 7.8 cm × 8.8 cm. The patient was treated with wide excision of the tumor. Core biopsy showed a metastatic adenosquamous carcinoma with suspected primary lesion from the respiratory tract. Treatment with targeted chemotherapy and radiotherapy were then done to the patient. The patient was discharged without any complications and is still at remission at the 6 months post-operative checkup.

**Clinical discussion:**

Soft tissue metastasis of lung cancer cell is a rare but a very real phenomenon. In our case the diagnosis of the soft tissue mass as a metastasis from the lungs was decided on a clinical, physical, radiological, and histological basis without using immunohistochemistry.

**Conclusion:**

MRI, biopsy, and immunohistochemistry are traditionally needed to confirm the diagnosis but in select cases, radiological and microscopic examinations along with clinical correlation are enough to ascertain the diagnosis. While it is rare, a soft tissue metastasis should always be suspected in lung cancer patients that have a palpable mass.

## Highlights

• Metastasis bone disease is one of the most common causes of bone malignancies,
• Metastasis to the muscle or soft tissue is rare
• Any soft tissue mass found in patient with lung cancer should warrant further examination
• Many factors are associated with musculoskeletal metastasis, but none have been established
• Established muscle metastasis can be treated with wide excisions of tumor

## 1 Introduction

One of the most common causes of bone malignancy is due to metastatic bone disease. It is estimated that 1 out of 5 patients with cancer will experience bone metastasis. More than 220.000 cases of non-small cell lung carcinoma are reported per year [1] but the prevalence of soft tissue metastasis from lung cancer is only 2.3 % making it commonly overlooked as a site of metastasis spread [1,2].

Lung cancer is one of the most common types of cancer encountered and the most common metastasis sites for lung cancers are the brain bone, live, and adrenal glands with the probability being 10 %, 7 %, 5 %, and 3 % respectively. Despite the hematogenous dissemination route of metastatic carcinoma, soft tissue metastases from lung cancer are generally rare with a prevalence of only 2.3 % [3,4].

Soft tissue metastasis is characterized as cancerous cell growth, either in the skeletal muscles or in the subcutaneous tissues following the diagnosis of the primary malignancy [5]. Skeletal muscle metastasis are similar to soft tissue sarcomas due to their clinical and radiological appearance [6].

The first reported lung metastasis to the muscle was done by Wittich in 1854 [7]. The only manifestation of muscle metastasis is a palpable mass near or at the muscle where the mass can be painful or painless, but it is more common for it to be painful. Around 83 % of the mass was found painful. Because of the subtle and uncommon finding, lung cancer metastasis to muscle should always be suspected when a palpable mass was found in examination because a late diagnosis might lead to an incorrect staging of cancer and leads to a delay diagnosis and appropriate treatment [3,5,8].

MRI can be used as a diagnostic modality, but a biopsy is still needed to make a definite diagnosis of muscle metastasis. After the diagnosis is made, the treatment should be adjusted according to the patient's age, global health status, tumor size, and overall severity of the disease. Surgical resection of the tumor has an overall better prognosis than conservative treatment.

This study is meant to report a case of a non-small cell lung carcinoma with an unusual metastasis to the soft tissue of the upper arm region [8].

## 2 Case report

A 54-year-old male presented to our outpatient clinic with a solitary and painful lump of the right upper arm. The lump has been getting bigger for the past 1 year and the pain has increased due to daily activities. The patient had no other symptoms. On physical examination, the tumor was felt on the right upper arm underneath the biceps muscle, approximately 8 cm × 8 cm in size, palpable as a solid mass, no warmth, and tender to palpation. Range of motion of the shoulder joint was limited due to pain. Upper extremity pulses and neurological examination were normal.

**Fig. 1:**
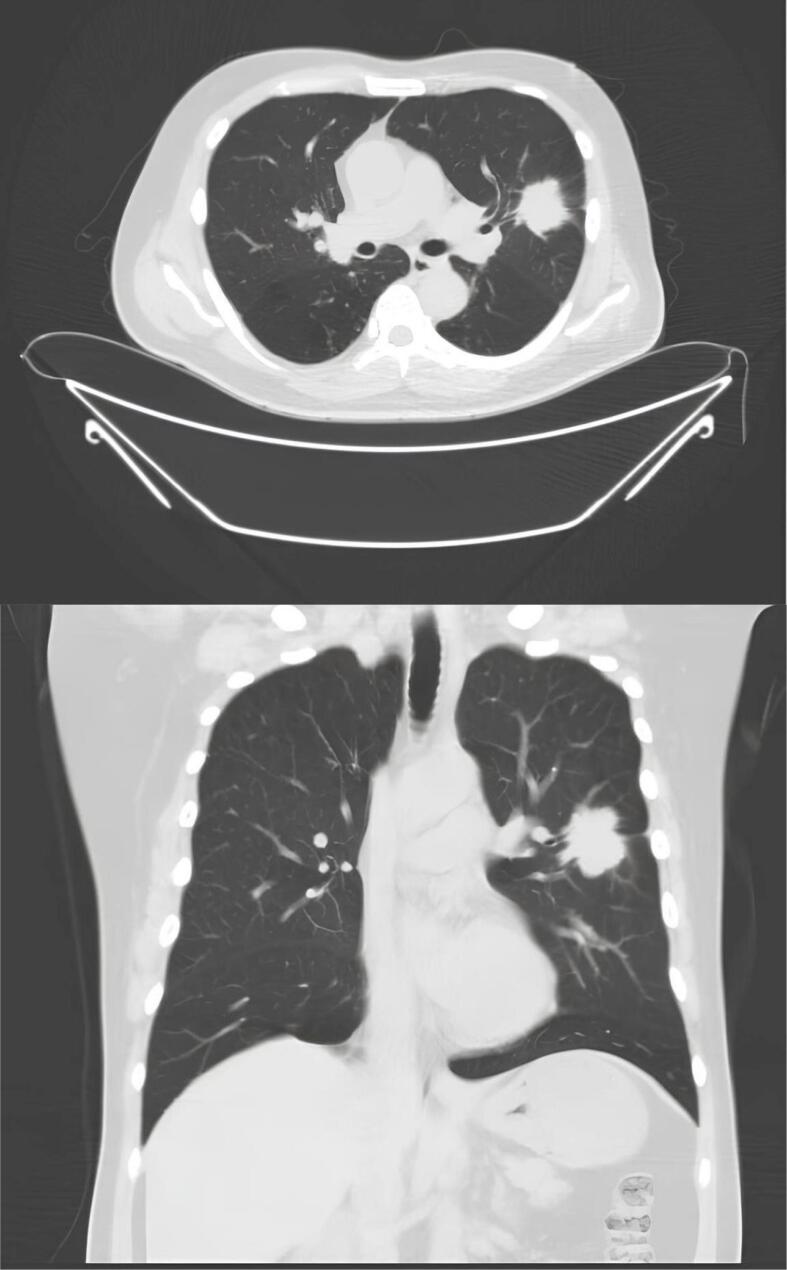
A homogeneous isodense lesion which is well demarcated and irregular borders with spiculae shadows.

CT scan of the lung showed a well demarcated, homogeneous isodense lesion with irregular borders and spiculae shadows that covers the anterior segment of the left superior lobe and superior lingula, approximately 3,4 cm × 3,2 cm × 2,3 cm in size and bronchiectasis on the supra-hilar area of the right superior and inferior lobe on both of the lungs [Fig. 1].

**Fig. 2:**
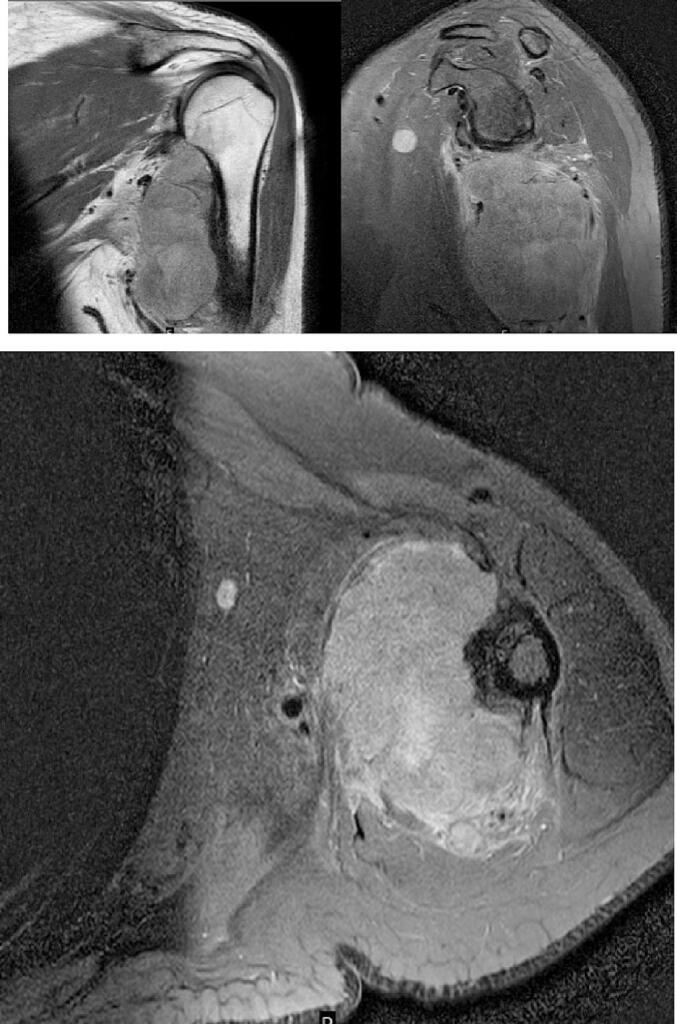
MRI of Left Shoulder: a dense soft tissue mass, lobulated, and indefinitely demarcated with size approximately 5,6 cm × 7,8 cm × 8,8 cm.

MRI of the right shoulder showed a 5,6 cm × 7,8 cm × 8,8 cm dense, lobulated, and indefinitely demarcated mass with involvement of the supraspinatus, biceps brachii, coracobrachialis, teres major, teres minor, latissimus dorsi, subscapularis, triceps brachii, and deltoid muscle. The soft tissue tumor eroded the scapula, the head and the diaphysis of the humerus. With lymphadenopathy of the left axilla, obliterating the suprascapular artery, vein, and nerve, with involvement of the axillary artery, vein, and nerve around the left humerus where the destruction resembles a malignant tumor. MRI revealed there was no involvement of the bone structure [Fig. 2].

**Fig. 3:**
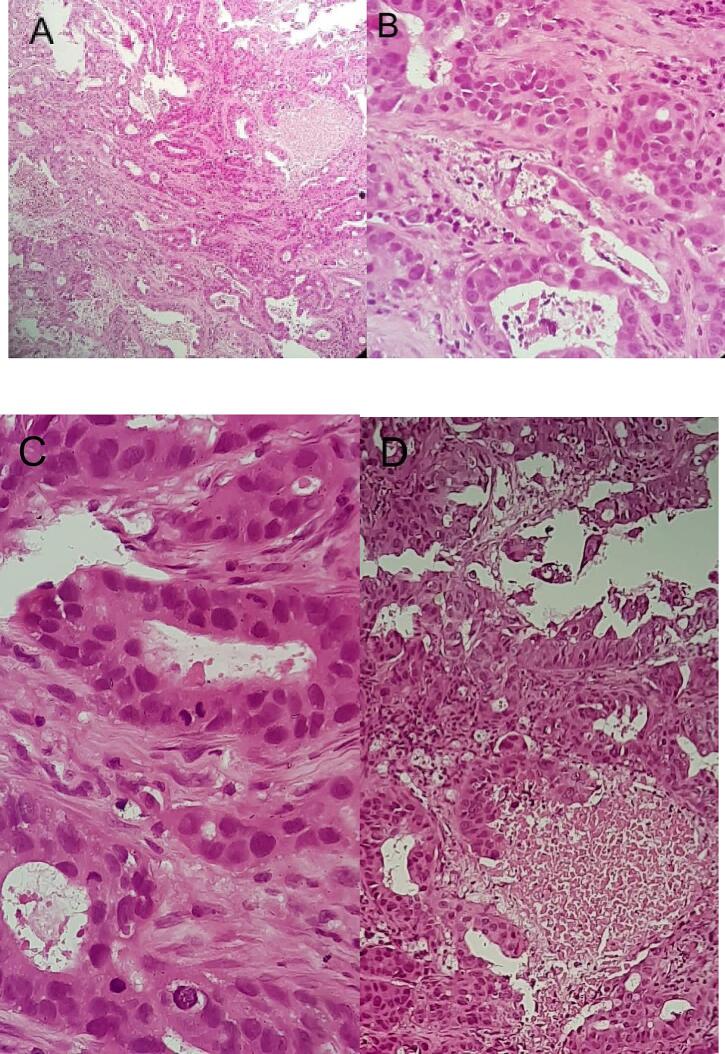
Histopathology examination. (A and B) Histopathology from the left lung. (C and d) Histopathology from the left arm.

A core biopsy was performed on the left arm. The result was a fibrous tissue which infiltrated with a massive tumor mass. Tumor masses form a structure proliferative gland, pleomorphic oval, tightly arranged and form a solid tumor nest. Tumor cell nucleus was large round oval, partially pleomorphic, rough chromatin, partially dense chromatin cells, and mitosis was found. The lumen of the gland is partially filled with necrotic tissue. Histopathological examination leads to metastatic adenosquamous cell carcinoma on the soft tissue of the left arm [Fig. 3].

## 3 Treatment

**Fig. 4:**
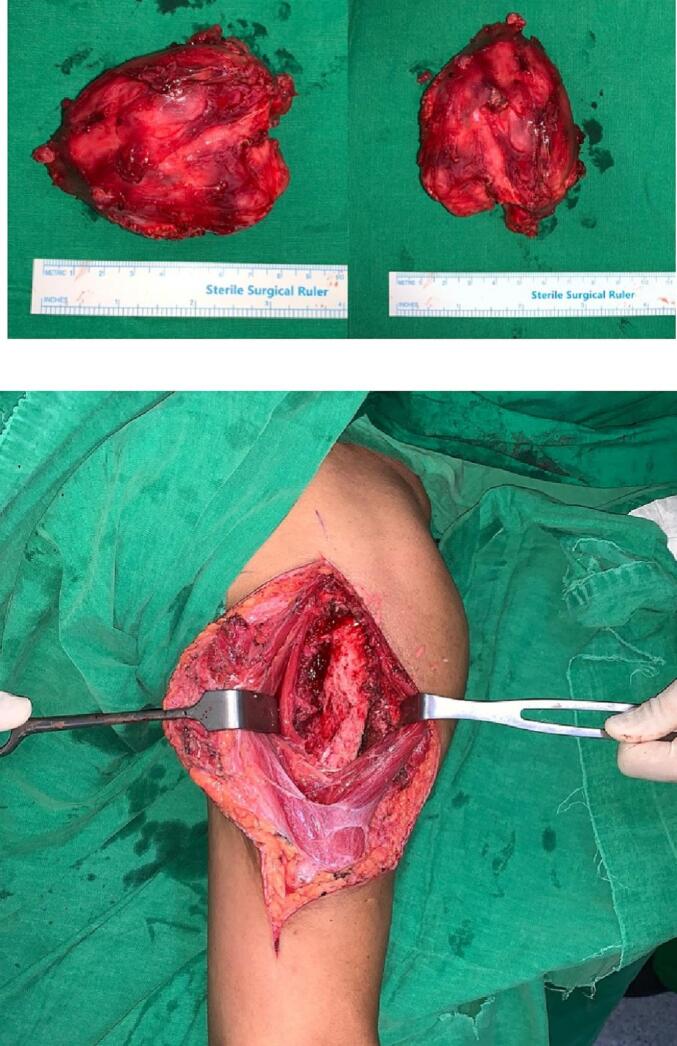
Soft tissue tumor, approximately 8 cm × 9 cm × 7 cm in size underneath biceps muscle.

After the diagnosis was established, the patient was treated with a wide excision from an anterolateral approach of the humerus. The incision was made on the anterior border of the deltoid muscle from a midway point between its origin and insertion and proceeded in line with the lateral border of the biceps muscle to within 7,5 cm from the elbow joint. Retract the deltoid muscle to the lateral side and biceps to the medial side to expose the soft tissue tumor. The tumor was found adhered to the surrounding tissue and the humeral bone. After removal of the entire soft tissue tumor, the size was approximately 8 cm × 9 cm × 7 cm [Fig. 4].

A core biopsy of the right upper arm tumor showed a fibrous collagen tissue arranged regularly, consisting of fibrous cells with a spindle nucleus, fine chromatin, and long eosinophilic cytoplasm. Among the connective tissue, there was an infiltration of tumor nests that were well-defined with their surroundings. Tumor nest consists of squamous-like epithelial cells, large nucleus, round, oval, hyperchromatic, strong eosinophilic cytoplasm. The core biopsy showed a malignant neoplasm with histopathological tissue in accordance to a metastatic adenocarcinoma. Which suggested that the primary tumor is in the lungs or respiratory tract. Further examination with immunohistochemistry to confirm the diagnosis was not done in this case because of limited resources and the combination of data from the clinical and collected data are deemed enough to decide on the diagnosis. After the diagnosis was decided, selective therapy and radiotherapy sessions were initiated. At control 6 months post operatively there is no recurrence of tumor but the patient was lost to follow up after the 6 months checkup. This work has been reported in line with the SCARE criteria [13].

## 4 Discussion

Our case represents a rare manifestation of a non-small cell carcinoma of the lung that metastasizes to extremity musculature. Various cases of soft tissue metastasis of tumor cells have been reported with varying amounts of end results and presentations^2.4^ One of them is a case by Baldeo et al., where a 54-years-old male with bilateral pulmonary nodules with an increasing mass in his right scapula. MRI Showed a scapular mass with internal necrosis and biopsy result was positive for metastatic adenosquamous carcinoma, favoring a primary lung carcinoma. The patient was treated with erlotinib and he passed away 6 months after diagnosis [4].

Although the skeletal muscle accounts for approximately half of total body mass, metastatic disease to the muscle remains an uncommon phenomenon in clinical practice. Almost all international publications are case reports and even larger case series of primary lung cancers with metastasis to the muscle do not exceed 10 cases [9]. In autopsy involving 500 lung cancer patients, Willis found 4 skeletal muscle metastasis [3]. Skeletal muscle metastasis commonly originated from tumors of the thyroid, esophagus, stomach, pancreas, colon, rectum, bladder, breast, ovary, and prostate [10].

The mechanism of the skeletal muscle metastasis is still unclear, but various mechanisms are hypothesized as a contributing factor to the metastasis mechanism, such as mechanical factors, metabolic factors, and immunological factors [6,7]. Weiss discovered that tumor cells injected into the muscle of a mouse have a harder time surviving in electrically activated muscles than in denervated or noncontractile control tissues. Muscle contractions can prevent metastatic cells from surviving within the tissue [11]. In the contractile state, blood flow is highly variable and is influenced by b-adrenergic receptors extrinsically. On the other hand, regular metastasis sites, such as the liver and bones, have high perfusion and steady blood flow. Muscle capillaries dilate during exercise, and the volume of blood supplied increases by up to 800 times as compared to resting state. Furthermore, mesenchymal tissues, such as skeletal muscle, contain diffusible proteases and other enzyme inhibitors that can inhibit enzyme-dependent invasion and tumor growth [7,12]. The metabolic hypothesis highlights the importance of local pH, lactic acid intake, and the generation of reactive oxygen species (ROS). Internal skeletal muscle characteristics such as elevated lactate output, pH instability, and variable oxygen tensions can create an environment which is unfavorable to the development of macroscopic tumor foci, rendering them undetectable [3,6,7]. Finally, the immunologic hypothesis focuses on cellular and humoral immunity such as the lymphocytes and the natural killer cells in the skeletal muscle [7].

In our case the diagnosis of the soft tissue mass as a metastasis from the lungs was decided on a clinical, physical, radiological, and histological basis without using immunohistochemistry. The authors are aware that immunohistochemistry is the preferred method for identifying and determining the specific types of unidentified tumor cells. But because there is already a suspected primary lesion in the lungs; as identified by CT-scan and compounded by the fact the from the microscopical slides of the tumor showed adenosquamous carcinoma, all of it correlate with each other and points to the direction of a lung metastasis. Also adding to the fact that there are limited resources in our facility that prevents us from easily doing the immunohistochemistry examination, we deemed that the diagnosis can be determined even without immunohistochemistry [14].

## 5 Conclusion

We report a rare case of a metastatic non-small cell lung carcinoma with an unusual metastasis as a soft tissue tumor in the muscle. MRI, biopsy, and immunohistochemistry are traditionally needed to confirm the diagnosis but in select cases, radiological and microscopic examinations along with clinical correlation are enough to ascertain the diagnosis. While it is rare, a soft tissue metastasis should always be suspected in lung cancer patients that have a palpable mass with, or without pain especially in patient with history of malignancy.

## CRediT authorship contribution statement

Dr. Andriandi H, M.ked (surg), sp.OT (K)

- Study concept
- Data analysis
- Writing the paper

Dr. Clement Tirta

- Data collection
- Data Analysis
- Writing the paper
- Study concept

## Declaration of competing interest

All authors deny any conflict of interest.
